# Can Control Infections Slow Down the Progression of Alzheimer’s Disease? Talking About the Role of Infections in Alzheimer’s Disease

**DOI:** 10.3389/fnagi.2021.685863

**Published:** 2021-07-22

**Authors:** Mingchao Shi, Chunrong Li, Xiaoping Tian, Fengna Chu, Jie Zhu

**Affiliations:** ^1^Department of Neurology, Neuroscience Center, The First Hospital of Jilin University, Changchun, China; ^2^Division of Neurogeriatrcs, Department of Neurobiology, Care Sciences and Society, Karolinska Institutet, Karolinska University Hospital, Solna, Sweden; ^3^Cognitive Impairment Ward of Neurology Department, The Third Affiliated Hospital of Shenzhen University Medical College, Shenzhen, China

**Keywords:** Alzheimer’s disease, infection, inflammation, treatment, microbiota

## Abstract

Alzheimer’s disease as the most common age-related dementia affects more than 40 million people in the world, representing a global public health priority. However, the pathogenesis of Alzheimer’s disease (AD) is complex, and it remains unclear. Over the past decades, all efforts made in the treatments of AD, with targeting the pathogenic amyloid β (Aβ), neurofibrillary tangles, and misfolded tau protein, were failed. Recently, many studies have hinted that infection, and chronic inflammation that caused by infection are crucial risk factors for AD development and progress. In the review, we analyzed the role of infections caused by bacteria, viruses, and other pathogens in the pathogenesis of AD and its animal models, and explored the therapeutic possibility with anti-infections for AD. However, based on the published data, it is still difficult to determine their causal relationship between infection and AD due to contradictory results. We think that the role of infection in the pathogenesis of AD should not be ignored, even though infection does not necessarily cause AD, it may act as an accelerator in AD at least. It is essential to conduct the longitudinal studies and randomized controlled trials in humans, which can determine the role of infection in AD and clarify the links between infection and the pathological features of AD. Finding targeting infection drugs and identifying the time window for applying antibacterial or antiviral intervention may be more promising for future clinical therapeutic strategies in AD.

## Highlights

- The infections caused by various pathogens have been evidenced to involve in the pathogenesis of AD; however, it is still controversial. Therefore, in-depth study of the causal relationship between infections and AD is very necessary.
- The infections may be one of the crucial pathogenic factors in development of AD through inducing neuroinflammation and aggravating the pathological changes, promoting the progression of AD.
- The treatments with anti-infections may slow down AD progression, but it needs to identify the time window for applying antibacterial or antiviral intervention, which might be a beneficial to AD patients.

## Introduction

Alzheimer’s disease is the most prevalent form of age-related dementia in the elderly population, and it has reported that about 40 million people suffered from Alzheimer’s disease (AD) in the world in 2017 (Levy et al., 2017), representing a global public health priority. The main pathological features of AD consist of amyloid-β (Aβ) plaque deposits, neurofibrillary tangles formed by hyperphosphorylated tau protein, and neuroinflammation, which are current major theory of the pathogenesis of AD (Angelucci et al., 2019). Despite a lot of intensive studies in the past, the pathogenesis and the etiology of AD remain largely unknown. Previous treatment of AD based on targeting the pathogenic Aβ or tau was unsuccessful, speculating that the pathogenesis of AD should be multifactorial and is more complex than a simple pathogenic Aβ or tau would suggest. Recently, several groups have demonstrated that infections or chronic inflammation caused by infectious agents are strongly involved in the pathogenesis of AD, which reignited interest in the infectious theory that was largely disregarded by AD research (Ashraf et al., 2019; Beydoun et al., 2020; Haditsch et al., 2020; Schnaider et al., 2020), since the role of infection in the development and progress of AD has long been debated and is still uncertain (Allnutt et al., 2020; Ou et al., 2020). Currently, the crucial role of infectious agents in AD-associated inflammatory pathways and antimicrobial properties of Aβ has been evidenced (Ashraf et al., 2019; Itzhaki et al., 2020).

Evidence based on the published studies showed that bacteria, such as *Helicobacter pylori* (*H. pylori*) (Malaguarnera et al., 2004; Kountouras et al., 2006; Albaret et al., 2020; Doulberis et al., 2020), and *Porphyromonas gingivalis* (*P. gingivalis*) (Singhrao et al., 2015; Haditsch et al., 2020), viruses, such as human immunodeficiency virus (HIV) (Lieberman, 2000; Smits et al., 2000; de Almeida et al., 2020), herpes simplex virus type 1 (HSV-1) (Cribbs et al., 2000; La Rosa et al., 2019; Linard et al., 2020), human herpes virus-6 (HHV-6) (Lin et al., 2002; Carbone et al., 2014), HHV-7 (Readhead et al., 2018), Epstein–Barr virus (EBV) (Xu and Gaskin, 1997; Gate et al., 2020), the novel coronavirus 2019 (COVID-19) virus that broke out in the end of 2019 (Ferini-Strambi and Salsone, 2020; Ou et al., 2020), *Chlamydia pneumoniae* (Arking et al., 1999; Itzhaki et al., 2020; Sundar et al., 2020; Woods et al., 2020), and *Borrelia burgdorferi* (Itzhaki et al., 2020), as well as fungi, such as *Saccharomyces cerevisiae, Malassezia globosa, Candida albicans* (*C. albicans*), and *Cryptococcus neoformans* (*C. neoformans*) (Alonso et al., 2014a, 2015; Pisa et al., 2015a; Carter, 2017; Santiago-Tirado et al., 2017), etc., pathogens, and the prion protein (PrP) (Snow et al., 1989; Come et al., 1993; Zhang B. et al., 2020), may be associated with development and progress of AD. Generally, it has a great possibility that infectious pathogens invading the brain may play a triggering role in AD development, supporting the existence of a direct or indirect nexus between infections/chronic inflammation caused by infections and AD.

In the review, we analyze the role and the underlying mechanisms of these infectious agents in the pathogenesis of AD in order to evidence targeting these infectious pathogens as a novel therapeutic manipulation in AD.

## Infections and Aging

It has been evidenced and is beyond doubt that aging results in increasing the severity and susceptibility to infections and diminishes ability to fight infection in aged people (Kim et al., 2015). The main reason is due to immunosenescence, which manifests as obviously decreasing immunoglobulin (Ig) diversity associated with declined number of mature B cells and circulating antibodies, weakening their response capacity to new antigens and impairing antibody function, including lowering affinities and opsonizing abilities during aging (Cicin-Sain et al., 2012; Derhovanessian et al., 2013). Also aged memory T helper (Th) 1-type autoreactivity results in age-related immune hyporeactivity in aged people and AD, whereas autoreactive memory Th2 cells could promote tumorigenesis (Pawelec, 2017; Seledtsov and von Delwig, 2020; Thomas et al., 2020), which is related to a harmful clinical outcome always (Pawelec, 2017; Dugan et al., 2020). In short, immunosenescence leads to immune dysfunction in aged people and AD (Pawelec, 2017; Dugan et al., 2020).

## Infections and Inflammation

To date, the published results have displayed the possibility that infection causes AD by creating inflammation (Soscia et al., 2010; Hur et al., 2020). Infections might cause a chronic, low-grade, sterile, and systemic inflammatory condition during aging called as “inflammaging” that has been recognized as a crucial pathogenic factor in the pathogenesis of AD. The features of inflammaging consist of enhancement of microglial activation and pro-inflammatory molecule production (Ponnappan and Ponnappan, 2011; Fulop et al., 2018), which deteriorates inflammation in aging people and AD (Abbas et al., 2002; Lambert et al., 2009).

There is growing evidence that reactive gliosis and microglial activation in AD brain, as the features of inflammation response, are the histological hallmarks and important factors in initial AD pathology (Salter and Stevens, 2017; Yeh et al., 2017; Zhang X. et al., 2020). Activated microglia in the central nervous system (CNS) are heterogeneous and categorized into two types: pro-inflammatory and anti-inflammatory microglia (Tang and Le, 2016; Yu et al., 2019), which play a dual role as either cytotoxic or neuroprotective roles, depending on the microglial phenotype and at the different stages of AD. The pro-inflammatory microglia can enhance inflammation and promote Aβ accumulation, inducing neural cell death and worsening disease through producing inflammatory mediators (Fakhoury, 2018; Zhang X. et al., 2020). Oppositely, Aβ plaques can be phagocytized by the anti-inflammatory microglia, which clean and degrade Aβ by increased phagocytic and lysosomal activity, as well as restriction of the inflammatory response (Kamphuis et al., 2016; Dubbelaar et al., 2018). We speculated that microglial over-activation or malfunction may be the basis of AD pathogenesis, which aggravates inflammation and accelerates the development of AD.

A large number of research results in the past showed that elevated levels of inflammatory cytokines/chemokines and mediators were associated with infections and inflammation in patients with AD and its animal models. Serum levels of pro-inflammatory cytokines/chemokines, including interleukin (IL)-6 (IL-6), CXCL2, and IL-1β, were higher in cognitive-impaired patients with brain amyloidosis than those without brain amyloidosis and controls; however, a significantly declined anti-inflammatory cytokine IL-10 was seen in the patients with brain amyloidosis compared with patients without brain amyloidosis (Cattaneo et al., 2017), implying pro-inflammatory cytokines/chemokines, contributing to brain amyloidosis and cognitive impairment. Additionally, it was observed a close relationship between higher levels of pro-inflammatory cytokines IL-1β and CXCL2 with bacterial infections (Cattaneo et al., 2017).

Similarly, the infections by *P. gingivalis* increased productions of inflammatory cytokines, such as tumor necrosis factor-alpha (TNF-α), IL-6, and IL-1β, as well as complement factors, nitric oxide (NO) and reactive oxygen species, which augmented Aβ deposits and activated complement system, causing inflammation, brain tissue degeneration, and cognitive impairment, consistent with the damage in AD (Boje and Arora, 1992; Floyd, 1999; Liccardo et al., 2020; Costa et al., 2021). Additionally, high levels of TNF-α, IL-1β, IL-6, IL-12, and granulocyte-macrophage colony-stimulating factor (GM-CSF) in plasma increased gut permeability in aging macaques, which was associated with leaky gut, inflammatory phenotype, and dysbiosis of gut microbiota, leading to AD and neurological disorders through a gut-brain axis pathway (Schneider et al., 2019; Sochocka et al., 2019).

Moreover, interferon (IFN) as an inflammatory boosting factor in the cultured brain cells enhanced levels of Aβ and IFN-induced transmembrane protein 3 (IFITM3) that can bind to γ-secretase, raising Aβ production, and act as a mediator in the process of inflammation and Aβ formation. When viruses invade into the brain, IFITM3 is activated and exercises its function (Hur et al., 2020). Furthermore, IFN-stimulated gene (ISG) signature was found in the brains of AD murine models and of wild-type mice, stimulated with generic nucleic acid-containing amyloid fibrils. Most convincingly, activated ISG-expressing microglia only surrounded nucleic acid+ Aβ plaques accumulated in an age-related way. In AD models, injection of IFN-β into brain caused microglial activation and blockading IFN receptor reduced microgliosis and synapse loss. The same phenomenon with activated ISG-expressing microglia with nucleic acid-containing neuritic plaques was observed in postmortem brains of AD. An IFN pathway in AD was obviously upregulated and clearly related to AD severity and inflammation (Devanand et al., 2020).

Previously, our study displayed that major inflammatory cytokines, IL-12, and IFN-γ in brain of transgenic mice APP_SWE_ (Tg2576) that overexpress the human Aβ precursor protein gene as an AD mouse model were associated with Aβ plaque formation and microglial and astrocyte activation (Abbas et al., 2002). In the brains of patients with AD and its animal models, infection stimulating microglia and/or microglial activation by peripheral cytokines caused robust production of free radicals with another wave of pro-inflammatory cytokines, which, in turn, led to inflammation and massive neuronal damage (Nakamura et al., 2021; Wiatrak and Balon, 2021).

Inflammation, playing a central role in the pathogenesis of AD, has reached consensus, while inflammatory mediators caused neuroinflammation through the damaged blood-brain barrier (BBB) entering to CNS. Thereby, BBB disruption was one of the determinant factors in development of AD, which has been evidenced by genetic, proteomic, and epidemiological study (Carter, 2017). So far, diverse pathogens have been detected in AD brains, indicating that infections may be related to the pathogenesis of AD. Disruption of BBB facilitated most pathogens to enter brain, which appeared an early pathological character of AD (van de Haar et al., 2016; Lecler et al., 2017). Siderosis and microbleeds in brain were also the features associated with BBB disruption in patients with (Yates et al., 2014; Inoue et al., 2016), since bacteria relied on the availability of free iron to assist in their successful colonization in AD.

It is noteworthy that infections not only cause acute diseases but also lurk in the body for a long period, leading to a chronic, low-grade, sterile inflammation and tissues damage, which is a key pathogenic factor in neurodegenerative diseases, such as AD (Chitnis and Weiner, 2017; Stephenson et al., 2018).

## The Role of Infections in AD and Its Animal Models

The infectious hypothesis of AD pathogenesis was highly overlooked previously due to lack of evidence. Recently, the infectious hypothesis of AD has been more and more recognized and accepted by the broad scientific community, since there are many new overpowering pieces of evidence of a close relationship between the infections by various pathogenic microbes and development of AD (Malaguarnera et al., 2004; Singhrao et al., 2015; Beydoun et al., 2020; de Almeida et al., 2020; Doulberis et al., 2020; Ferini-Strambi and Salsone, 2020; Gate et al., 2020; Haditsch et al., 2020; Linard et al., 2020). It is often seen that patients with mild cognitive impairment (MCI) or early dementia, suffering from infection, manifested a speedy decline in cognitive function and could not completely recover after therapy of infections. It is possible that persistent infection worsens gradually cognitive function (Itzhaki et al., 2020). It has been proved that *P. gingivalis* infiltration was found in autopsy specimens from brains and in cerebrospinal fluid (CSF) of patients with AD (Laugisch et al., 2018; Ryder, 2020). Moreover, it has been observed that the etiology and pathologies of AD may be related to the infections by several bacteria, viruses, and fungi, as well as other pathogens, which support that infections are a risk factor and play a crucial role in AD (Snow et al., 1989; Come et al., 1993; Xu and Gaskin, 1997; Cribbs et al., 2000; Lieberman, 2000; Smits et al., 2000; Lin et al., 2002; Malaguarnera et al., 2004; Kountouras et al., 2006; Alonso et al., 2014b, 2015; Carbone et al., 2014; Singhrao et al., 2015; La Rosa et al., 2019; Albaret et al., 2020; de Almeida et al., 2020; Doulberis et al., 2020; Gate et al., 2020; Haditsch et al., 2020; Linard et al., 2020; Zhang B. et al., 2020).

### Infections by Various Pathogens in AD and Its Animal Models

#### Bacterial Infections

Bacterial infections, mainly referring to the infections with *P. gingivalis* and *H. pylori*, have been shown to be linked to AD, which is definitive evidence, proving the infection associated with AD so far (Malaguarnera et al., 2004; Kountouras et al., 2006; Laugisch et al., 2018; Beydoun et al., 2020; Liccardo et al., 2020; Ou et al., 2020).

##### *P. gingivalis* infections

*Porphyromonas gingivalis* and other periodontal pathogens can penetrate the bloodstream and enter the brain, inducing a low-grade systemic inflammation that negatively affects brain function (Liccardo et al., 2020). These periodontal pathogens were found in periodontium, serum, and CSF of AD. However, periodontal pathogens could not trigger development of AD in the demented patients younger than 70 years old (Laugisch et al., 2018). The antibodies to periodontal bacteria, including *Aggregatibacter actinomycetemcomitans*, *P. gingivalis, Campylobacter rectus*, *Treponema denticola*, *Fusobacterium nucleatum*, *Tannerella forsythia*, and *Prevotella intermedia*, were enhanced obviously in the patients before cognitive impairment, indicating that periodontal bacteria are key risk factors in AD occurrence (Sparks Stein et al., 2012). The infected neurons by *P. gingivalis* appeared the similar signs to AD neuropathological changes, for example, increased in the phospho-tau/tau ratio, synapse loss, etc. (Haditsch et al., 2020), supporting the existence of a direct nexus between periodontitis and AD. The gingipains, a class of *P. gingivalis* proteases, are related to neuronal damage, tau tangles, and Aβ deposit in the brains of patients with AD (Ryder, 2020). Additionally, the brain of the patients with AD can be infected by oral pathogens and their toxic products. During the period of the disease progress, patients with AD are unable to adequately maintain oral hygiene, resulting in oral infections and periodontitis; in turn, it deteriorated AD forming a vicious circle (Liccardo et al., 2020; Sadrameli et al., 2020). A diminished level of salivary lactoferrin, a credible biomarker for Aβ accumulation in AD brains, has been found more recently, because a lower level of salivary lactoferrin altered the oral microbiota, which was an outcome of poor oral hygiene and dry mouth in the elderly and promoted oral bacteria to grow and to spread elsewhere in the body (Nie et al., 2019). Oral bacteria could grow locally and followed the infected inflammatory monocytes/macrophages in the mouth, spreading elsewhere in the body through the bloodstream or these bacteria penetrated the bloodstream directly to reach various parts of the body, in which the activated cathepsin (Cat) B (CatB)/nuclear factor kappa-B (NF-κB) signaling pathway by bacteria may contribute to the spreading process (Nie et al., 2019). The pathogens of periodontal disease causing chronic inflammatory responses impact significantly on the development of AD. The evidence showed that microbial infections and chronic inflammation in the brain may be involved in the pathogenesis of AD (Angelucci et al., 2019; Ashraf et al., 2019; Beydoun et al., 2020; Haditsch et al., 2020).

In mice, oral infections with *P. gingivalis* showed *P. gingivalis* infiltration into brain, accompanying with various neuropathological features of AD and exacerbated disease (Nie et al., 2019). *P. gingivalis* as an oral pathogen can entry into apolipoprotein (APOE) knockout (KO) (equivalent to APOE 4 genotype) brain of mice to induce complement activation (Poole et al., 2015). Although there are various means of entry for periodontal bacteria to access brain (Riviere et al., 2002), *P. gingivalis* entered APOE KO brain of mice mainly through blood circulation and circumventricular organs (Velsko et al., 2014; Poole et al., 2015; Ilievski et al., 2018). Also *P. gingivalis* was able to access APOE KO brain of mice *via* other routes, including a permeable BBB affected by *P. gingivalis* or the choroid plexus, containing a rich blood supply and CSF (Riviere et al., 2002). Particularly, it is very easy for *P. gingivalis* accessing APOE KO brain of mice due to APOE deficiency, leading to a BBB leakage (Hafezi-Moghadam et al., 2007). Treatment with the compounds that inhibit *P. gingivalis* or its toxins in infected mice reduced the abundance of *P. gingivalis* DNA in brain and ameliorated the pathologies and the neurotoxic effects of *P. gingivalis* infection, which implies that control of *P. gingivalis* infection can affect the pathological condition of AD (Matsushita et al., 2020; Olsen and Singhrao, 2020), and the inhibitors of *P. gingivalis* provided a potential approach to treat both periodontitis and AD (Ryder, 2020). In addition, chronic systemic *P. gingivalis* infection led to the accumulation of Aβ in the brain, the most significant pathological hallmark of AD, in middle-aged mice, and also induced Aβ accumulation in inflammatory monocytes/macrophages through activating CatB/NF-κB signaling, indicating that monocytes/macrophages act as a circulating pool of Aβ in patients with periodontitis (Nie et al., 2019). It is worth noting that repeated oral infection by *P. gingivalis* caused neurodegeneration and an extracellular Aβ42 deposit in brain of young adult wild-type (WT) mice, which displayed that low-grade chronic periodontal pathogen infection led to occurrence and development of AD-like neuropathology (Ilievski et al., 2018). These results from cross-sectional and longitudinal studies showed that periodontitis is closely associated with cognitive impairment and AD.

The exact molecular mechanisms behind the infections by *P. gingivalis* and periodontal disease contributing the pathogenesis of AD remain unclear; however, it has been accepted, although not confirmed completely, that oral pathogens can infect and shift to the brain, inducing a low-grade local and systemic inflammation and affecting brain functions.

##### *H. pylori* Infections

*Helicobacter pylori* infections are a common gastrointestinal disease, leading to gastrointestinal dysfunction always. AD and cognitive decline, as well as other neurodegenerative diseases, are often associated with gastrointestinal dysfunction (Zhuang et al., 2018; Sochocka et al., 2019; Ticinesi et al., 2019). Levels of IgG and IgA antibodies to *H. pylori* were detected in 30 patients with AD, 30 patients with vascular dementia (VaD), and 30 control subjects, respectively, in a study (Malaguarnera et al., 2004). The results exhibited that significant high *H. pylori*-IgG levels were seen in AD when compared with normal controls but lower than those of patients with VaD. *H. pylori*-IgA levels were equally enhanced in patients with VaD and AD, which found a relationship between *H. pylori* infection and AD (Malaguarnera et al., 2004). Another study investigated the association between *H. pylori* infection and AD by using histology for diagnosis of *H. pylori* in total of 50 patients with AD and 30 iron deficiency anemic controls without AD. The results showed high histologic prevalence of *H. pylori* with 88% in AD and 46.7% in controls; the comparison between the two groups was very significant (*p* < 0.001) (Kountouras et al., 2006). More recently, a systematic review article has reported that the most common investigated microbiota was *H. pylori* in 241 published papers on a connection between AD and gastrointestinal microbiota, and displayed an association between AD and *H. pylori* (Doulberis et al., 2020). The risk of *H. pylori* infections in AD and Parkinson’s disease (PD) from the obtained data revealed increase in the risk of developing AD (Fu et al., 2020), indicating AD clearly associated with intestinal disorders, especially intestinal infections by *H. pylori* (Fu et al., 2020). *H. pylori* infection was associated with a low mini-mental state examination (MMSE) score in patients with AD, possibly *via* triggering the inflammatory mediator productions (Roubaud-Baudron et al., 2012) and increasing levels of Aβ40 and Aβ42 in serum of patients with AD (Bu et al., 2015). *In vitro*, the neuroblastoma cell cultures treated by *H. pylori* filtrate induced tau hyperphosphorylation, which was similar to AD tau pathological changes (Wang et al., 2015). Infected aged C57BL/6 mice with *H. pylori* or *Helicobacter felis* or left uninfected for 18 months appeared severe gastritis and increased neuroinflammation in infected groups (*H. felis* > *H. pylori*), but without brain Aβ deposition or systemic inflammation (Albaret et al., 2020).

The onset of AD and all-cause dementia can be changed by coinfection with *H. pylori* and the groups of periodontal pathogens, including *P. gingivalis*, which was evidenced *via* investigating 1,431 participants (≥65 years), with a mean follow-up of 10–11 years for AD and an all-cause dementia incidence (Beydoun et al., 2020). Epidemiological and clinical studies have shown that infections with *H. pylori* increased the risk of developing AD. However, the questions are whether this association is due to the ubiquity of *H. pylori*, or whether it promotes neuroinflammation in AD. The relationship between *H. pylori* and AD, if confirmed by additional studies with a large sample of patients, might have important clinical and therapeutic implications.

#### Virus Infections

Alzheimer’s disease is a multifactorial disease caused by both environmental factors and a genetic predisposition.

With the discovery of bacteria, viruses, and other pathogens in the brain of patients with AD, more and more evidence shows that AD is caused by microbial infection and inflammation (Jamieson et al., 1991; Kountouras et al., 2006; Readhead et al., 2018). Besides bacteria, the infections with viruses also contribute to the pathogenesis of AD. The viruses linked to development of AD include HIV, HSV-1, HHV-6, HHV-7, EBV, hepatitis C, and COVID-19, as well as Torque teno virus (TTV), which are gradually being recognized as important factors affecting the development of AD.

##### HIV Infections

In the patients with acquired immune deficiency syndrome (AIDS), infected by HIV, manifested cognitive deficits or dementia as the age-related complications, following patients living longer after treatments. Aβ deposits have been observed in patients with AIDS, which may be associated with HIV infection-induced neuroinflammation (Canet et al., 2018; Hategan et al., 2019; Kodidela et al., 2019). Recently, it has been found that the connection between HIV and AD could be extracellular vesicles (EVs)/exosomes, because they are able to package and transport the toxic proteins in both AD and HIV (Aβ/tau and gp120/tat, respectively) (Kodidela et al., 2019). Elevated Aβ in neuron-derived exosomes from the plasma of patients with AIDS showed neuron-to-neuron exosomal transport of Aβ and tau also contributing to AD-like pathology in the patients with AIDS (Kodidela et al., 2019).

Human immunodeficiency virus passing BBB causes neuronal dysfunction, leading to cognitive impairment. In the process of HIV infections, many factors involved in AD pathology, such as members of Aβ and tau pathways, are regulated by HIV infection, resulting in the accumulation of Aβ plaques or neurofibrillary tangles (NFT) in brains, indicating the similarities and convergences of the two pathologies in AD- and HIV-infected brains. HIV and its proteins, such as *trans-*activator of transcription (Tat) and envelope glycoprotein gp120, can modulate cellular pathways to deteriorate AD (Canet et al., 2018). HIV-Tat interacts also with Aβ and tau *via* novel a protein-protein coaction and induces tau phosphorylation through a cascade of cellular processes to form NFT, eventually resulting in AD, despite adequate antiretroviral therapy (Hategan et al., 2019). HIV-Tat may accelerate AD-like pathology in patients with HIV. There is a double risk in the older patients infected with HIV, those who may suffer from both HIV-associated neurocognitive disorder (HAND) and AD, which has been evidenced by investigating neuroimaging and neuropsychological performance in 61 older adults with HAND, 53 with MCI of a type of AD and 89 healthy controls cross-sectional over 20 months (Matsushita et al., 2020).

HIV-associated neurocognitive disorder as a cognitive disorder is similar to AD, and its pathogenesis involves the effects of HIV and its proteins, such as Tat and gp120 by destroying neurons and initiating neuroinflammation contributing to the deposition of Aβ and leading to dementia. HIV infection is associated with increased Aβ deposits in brain, a feature of AD (Fulop et al., 2019). Thus, the questions arise whether HAND may lead to AD or is it a form of AD? These questions are needed to further explore and study.

##### HSV Infections

A study that has followed more than 8,000 people with HSV infection and 25,000 healthy controls in Taiwan for 10 years found that increased risk of developing AD in patients with HSV was 2.5-fold higher than that in controls (Tzeng et al., 2018). Recent study has emerged that anti-herpetic drugs play a protective role in dementia through declining the risk of dementia in patients with HSV infections (Tzeng et al., 2018).

###### HSV-1 infections

Infection with HSV-1 is very common, and more than half of the world population hide it in their bodies. As early as 1990, HSV-1 was observed in postmortem AD brains (Jamieson et al., 1991), which evidenced the relationship between HSV-1 infection and AD for the first time. However, someone questioned that HSV-1 or other microbes were able to leak into brains easily after death due to tissue of brain breaks down; therefore, the pathogenesis of AD basis on postmortem material was an assumption (Jamieson et al., 1991).

A study on evaluating the impact of HSV-1 infection on the incidence of AD in the light of APOE4 status, a genetic susceptibility factor for AD, has been conducted *via* estimating 10-year risk of AD associated with anti-HSV antibodies in 1,037 subjects by Cox models (Linard et al., 2020). The results showed that frequency of HSV-1 reactivation was very high in APOE4 carriers manifested as raised an HSV-1 IgG antibody accompanied by increased risk of AD (Linard et al., 2020), suggesting a role of HSV-1 in the pathogenesis of AD in populations with a genetic susceptible factor, the APOE4 allele.

There is growing evidence that HSV-1 is gradually recognized as an important factor impacting the occurrence and development of AD. However, the mechanism linking HSV-1 and AD remains unclear. New research finds that HSV-1 manipulates the activity of cofilin-1 involved in the plastic of dendritic spines and axon regeneration of neurons to ensure HSV-1-infecting neurons efficiently. Dysfunction of cofilin-1 was observed in patients with AD and its animal models. The cognitive impairment in AD models can be improved by administration of inhibitors of cofilin-1 (Wang et al., 2020). HSV-1 infection-causing dysregulation of cofilin-1 led to AD finally probably. Pathologically, Aβ and hyperphosphorylated tau accumulations were observed in the neurons infected by HSV-1 and bacterial ligands; viral and bacterial DNA and RNA can increase the expression of pro-inflammatory molecules (Ashraf et al., 2019). To this, very strong evidence is that, after HSV-1 was injected into the brains of young 5xFAD mice, transgenic mice of AD, and of normal mice in 3 weeks, the brains of transgenic mice were covered fully with Aβ plaques (Eimer et al., 2018).

##### HHV-6 Infections

The role of HHV-6 in AD has been controversial, and recent studies have renewed the debate again. Previously, it was reported that HHV-6 existed in 10% of healthy brains in humans and mice; therefore, humans were not susceptible to HHV-6 infection (Eimer et al., 2018). But more recent study has demonstrated that adding HHV-6 to the human neural cell cultures that simulated pathologies of AD led to Aβ-plaques and tau tangles accumulation after 2 days compared with the cultures without HHV-6 after cultures for 6 weeks (Eimer et al., 2018), hitting that HHV-6 infection-accelerated Aβ-plaques and tau tangles accumulation, the two main pathological features of AD.

Human herpes virus-6 as neurotropic virus can dysfunction autophagy and activate endoplasmic reticulum (ER) stress/unfolded protein response (UPR) *in vitro* cell lines (Romeo et al., 2020), which is necessary for cell homeostasis, particularly in postmitotic cells, such as neuronal cells. Autophagy dysregulation and abnormal UPR activation are always observed in AD and other neurodegenerative disorders. A recent study has evidenced that primary neurons infected by HHV-6A decreased obviously autophagy, increased Aβ deposition, and activated ER stress/UPR, promoting tau protein hyper-phosphorylation. The levels of HHV-6A and HHV-7 were higher in AD than in controls by detecting the snippets of genes or proteins specific to herpes (Readhead et al., 2018; Romeo et al., 2020). These results showed HHV-6A infection playing a role in AD (Romeo et al., 2020).

However, a recent experimental result has negated the above conclusion and considered no association between HHV-6 and AD through screening for HHV-6 across three independent AD brain repositories, using (1) RNA sequencing (RNA-seq) datasets and (2) DNA samples extracted from AD and non-AD control brains. These direct methods of viral detection did not support HHV-6 playing a role in AD (Allnutt et al., 2020). The proving causality between HHV-6 and AD has been elusive, and more studies are needed to conduct in the future. Fortunately, valacyclovir, an antiviral drug started to treat mild AD with HSV antibodies together in 2017 and will be completed in 2022. If the clinical trial is successful, it will provide a proof that HHV can increase cognitive impairment and participate in the development of AD.

##### COVID-19 Virus Infections

Currently, the global epidemic of COVID-19 infection has potential implications for the field of AD, because COVID-19 caused severe respiratory symptoms in people aged 60 years and older; it has important implications for patients with AD. However, the exact impact of COVID-19 infection on AD is still uncertain due to lack of long-term follow-up and research. According to the published data, the preliminary conclusions concerning the effects of COVID-19 virus infections on AD are inconsistent (Brown et al., 2020; Fotuhi et al., 2020; Li et al., 2020; Naughton et al., 2020; Yu et al., 2020). Whether COVID-19 increases the risk and the incidence rate of future dementia is still to be further studied.

The clinical evidence showed that COVID-19 infection can cause acute neurological symptoms, such as a headache, confusion, epilepsy, delirium, and encephalopathy, as well as loss of taste and smell, etc., it also develops future diseases as long-term consequences of COVID-19 infection, such as depression, insomnia, cognitive decline, accelerated aging, PD, or AD in the future (Ferini-Strambi and Salsone, 2020; Fotuhi et al., 2020). It has been corroborated the presence of COVID-19 in the brain tissue and CSF of the infected patients by electron microscopy, immunohistochemistry, and real-time reverse transcription-PCR (Netland et al., 2008; Baig et al., 2020). COVID-19 infection can induce neuroinflammation, a prominent characteristic in AD pathology, and immune response in brain, which accelerates brain inflammatory neurodegeneration, and the elderly people are more susceptible to severe outcomes after COVID-19 infection. The patients with type 2 diabetes increased a risk for development of AD after COVID-19 infection (Bostanciklioglu, 2020; Fotuhi et al., 2020; Naughton et al., 2020). The epidemic has restricted activities and aggravated AD as a previous study showed movement restriction exacerbating AD symptoms (Lin et al., 2015). Higher rates of deaths of COVID-19 infection have been reported in older patients, who had comorbidities, such as hypertension, cardiovascular disease (CVD), and dementia (Vallamkondu et al., 2020). However, a report from Wuhan city of China showed, when compared with patients with non-AD, patients with AD had a better prognosis after COVID-19 infection due to the patients with AD had full access to health care (Li et al., 2020). In addition, a study that tested 13,338 cases for COVID-19 in United Kingdom showed that the patients with AD had highest risk of COVID-19 infection and mortality than normal elderly. However, the patients with PD enhanced risk of COVID-19 infection, but not mortality from COVID-19, suggesting that there was different susceptibility to COVID-19 in patients with neurodegenerative disorders (Yu et al., 2020). Patients carrying APOEε4 infected with COVID-19 had obvious odor recognition deficits when compared with those not carrying APOEε4, speculating that COVID-19 infection may increase the risk of dementia in APOEε4 carriers in the future (Ferini-Strambi and Salsone, 2020). Generally, the impact of COVID-19 on AD is age rather than neuropathology itself, whereas neuropathology rather than age may be critical in neuroimmune diseases, such as multiple sclerosis (Ferini-Strambi and Salsone, 2020).

##### EBV Infections

Epstein–Barr virus has been reported to associate with AD pathogenesis, and it was presented as two separate EBV antigens in CSF of patients with AD (Gate et al., 2020). EBV-transformed B cell lines from the peripheral blood of the patients with AD produced anti-Aβ antibodies in Aβ plaques of AD (Xu and Gaskin, 1997). Besides the descripted infectious pathogens as the above, recently, some researchers have demonstrated that the respiratory bacteria *Chlamydia pneumoniae* may be also implicated in AD, because the infection with *Chlamydia pneumoniae* has been associated with dementia, cortical atrophy, and pathological and biological hallmarks of AD (Ashraf et al., 2019). However, the results remain still controversial (Taylor et al., 2002; Boelen et al., 2007; Woods et al., 2020). Therefore, further studies are required for substantiation of causality and for the discovery of promising novel therapies.

These data further demonstrated that infections and their causes of chronic inflammation may be associated with developing AD. Nevertheless, it is still elusive whether immune dysfunction in AD brains reactivates latent viruses to prompt neurodegeneration or these viruses themselves trigger the neurodegenerative process in AD. Even though the microorganisms do not necessarily cause AD, they may act as accelerators in AD at least. This association does not yet prove a causal link, but these results call to conduct more studies on the microbial infection-AD hypothesis to determine whether therapeutic strategy targets infectious pathogens, which can slow down AD progress and is viable in the future (Ou et al., 2020; Schnaider et al., 2020).

Nonetheless, the infection hypothesis has been challenged in the pathogenesis of AD. The special issue is lack of experimental and preclinical evidence, showing that pathogens can cause directly neuropathological changes and cognitive impairment, which are the main characteristics of AD. Several studies have demonstrated that the transmissibility of AD neuropathology or cognitive impairment could not be observed after injection of AD brain homogenate directly into the brain of primates (Ou et al., 2020; Schnaider et al., 2020). There is still insufficient evidence to prove the existence of invaded intact virions or viral proteins in AD brain (Lellouch-Tubiana et al., 2010). Thus, infection associated with the etiology of AD may be unconvincing (Ou et al., 2020; Schnaider et al., 2020). Additionally, there was no significant difference between patients with AD and the controls for tested *Chlamydia pneumoniae* infection, which may conclude *Chlamydia pneumoniae* infection did not play a role in the etiology of AD (Campbell et al., 1998).

#### Fungal Infections

After providing extensive evidence by several research groups, the fungi infections are considered to be causative agents and risk factor in AD unequivocally on the basis of detected diverse fungi in AD brains, such as *Saccharomyces cerevisiae*, *Malassezia globosa*, *C. albicans*, *C. neoformans*, *Malassezia restricta*, *Cladosporium*, *Neosartorya hiratsukae*, and several other *Candida* species (Alonso et al., 2014a,b, 2017; Pisa et al., 2015a,b; Carter, 2017). Another piece of irrefutable evidence is that fungal DNA and proteins were found in CSF and peripheral blood of patients with AD (Alonso et al., 2014b, 2015). Besides, there were fungal components in postmortem brain tissues of patients with AD, and several peptides clearly corresponding to fungal proteins were observed by proteomics analysis (Alonso et al., 2014a; Pisa et al., 2015b). Strikingly, yeast-like cells and hyphal structures in AD brain were observed directly, which were related to neural cells both intra- and extracellularly (Pisa et al., 2015a,b). Additionally, increased levels of chitinase, a component of fungi and an inflammatory marker, were detected in blood and CSF of patients with AD, even before the onset of AD, which is a further support for the viewpoint that mycoses are present in AD (Rosén et al., 2014; Melah et al., 2016).

In patients with AD, *Alternaria*, *Botrytis*, *Candida*, and *Malassezia* were prevalent fungal genera analyzed by the group of Alonso (Alonso et al., 2018). Fungi detected from frontal cortex samples of patients with AD emerged clustered together and differed from the controls (Alonso et al., 2018). It is worth noting that coinfections with fungi and bacteria together were found in patients with AD, implying polymicrobial infections existing in AD (Carter, 2017; Alonso et al., 2018).

The cell surface amyloid-like structures in the pathogenic fungi are for adhesion and biofilm formation. An *in vitro* study, binding of the human constitutive plasma protein serum amyloid P component (SAP) to the surface amyloid of fungi inhibited immune response to fungi and diminished the phagocytosis of fungi by macrophages, there by SAP binding to fungi may help fungi infections in AD (Behrens et al., 2019).

In short, these observations support the hypothesis that the existence of fungal infections in patients with AD may be associated with the pathogenesis of AD (Pisa et al., 2015b; Alonso et al., 2017, 2018). However, the same fungi could not detect in all AD brains, or in different laboratories; therefore, further studies are required to confirm certain fungi in patients with AD.

#### Prion Protein (PrP) Infections

Accumulation of the pathological prion protein, scrapie prion protein (PrPSc), results in the fatal prion diseases. Cellular prion protein (PrP^C^) is also involved in other neurological disorders, including AD (Snow et al., 1989; Come et al., 1993; Zhang B. et al., 2020). There are protein misfolding and aggregation in both AD and prion diseases, which are underlying mechanisms of the diseases (Kodidela et al., 2019). It is clear that PrP^C^ plays a crucial role in AD development. Infections led to Aβ deposition and increased PrP^C^ and then caused high affinity binding between Aβ oligomers and PrP^C^, finally activated the Fyn signaling cascade, resulting in neuron death in brain of AD (Guerrero-Muñoz et al., 2014; Kodidela et al., 2019; Zhang B. et al., 2020).

**FIGURE 1:**
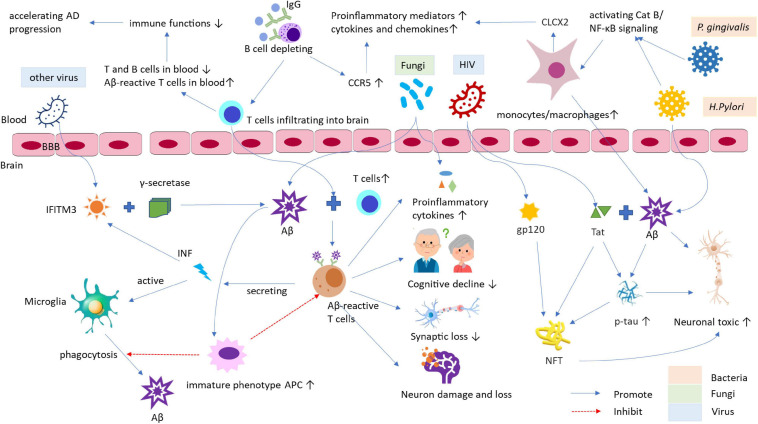
The role of infection and inflammation in the pathogenesis of AD and its animal models. The infections by bacteria, viruses, and other pathogens, and it caused chronic inflammation may be the crucial risk factors for AD development and progress. These pathogens can access into the brain through increased permeability of BBB. Afterward, the viruses active IFITM3 can bind to γ-secretase raising Aβ accumulation and deposition in brain and act as a mediator in the process of inflammation and Aβ formation. The bacteria, such as *P. gingivalis* and *H. Pylori*, can induce Aβ accumulation in inflammatory monocytes/macrophages through Cat B/NF-κB signaling. Activated monocytes/macrophages secrete CXCL2 can increase productions of proinflammatory mediators and cytokines/chemokines, leading to the pathological changes being similar to those of AD, for example, the elevated phospho-tau/tau ratio and synapse loss, etc. HIV and its proteins, such as Tat and envelope glycoprotein gp120, can modulate cellular pathways to deteriorate AD. HIV-Tat can interact with Aβ and tau *via* novel protein-protein coaction and induces tau phosphorylation through a cascade of cellular processes to form NFT, eventually resulting in neuronal toxicity. Additionally, fungi can also enter the brain to enhance levels of proinflammatory cytokines and bind to SAP to diminish the phagocytosis of macrophages, which cause Aβ accumulation, exacerbating AD. Furthermore, infection and inflammation can decline immune function and response contributing the pathogenesis of AD. AD, Alzheimer’s disease; APC, antigen-presenting cell; Aβ, β-amyloid; BBB, blood-brain barrier; Cat, Cathepsin; CCR5, C chemokine receptor type 5; CNS, central nervous system; CXCL2, chemokine ligand 2; HIV, human immunodeficiency virus; IFITM3, IFN-induced transmembrane protein 3; IFN, interferon; NFT, neurofibrillary tangles; NF-κB, nuclear factor kappa B; p-tau, phospho-tau; SAP, serum amyloid P component; Tat, *trans-*activator of transcription.

Cellular prion protein can act as a neurotoxic protein, and Aβ oligomer in AD has been found as a ligand of PrP^C^ that is critical to Aβ_42_-mediated autophagy in neurons. However, the role of PrP^C^ as an Aβ_42_-binding protein is not clear (Onodera, 2017). The role of miRNA-146a among all studied microRNAs is age-related and associated with human inflammatory neurodegenerative disease, including AD and prion diseases (Lukiw, 2020). Infection and inflammation hypothesis of AD pathology is summarized in Figure 1.

## Can Control Infections Be a Therapeutic Manipulation in AD?

Alzheimer’s disease has been considered as a systemic disease related to infections, and the inflammatory-infectious hypothesis of its pathogenesis becomes more significant (Bronzuoli et al., 2016). The microbes and their products in the brain causing chronic inflammation are an important predisposing factor in neuroinflammation and neurodegeneration in AD (Cattaneo et al., 2017; Ashraf et al., 2019). Thus, a proper combination of anti-inflammatory, antibiotic, and antiviral therapeutics might potentially prevent the development and progression of AD, even though there was much disappointment with anti-AD drug discovery previously, it is still promising and possible to find a new-treatments basis on infections impacting on AD.

The causal relationship between infection and AD remains uncertain and controversial; however, there is a possibility of reversed causality. Generally, patients with AD have poorer general health, and their ability and response of innate and adaptive immune are low, resulting in more likely to have infections (Shi et al., 2021). That is, infection may trigger AD, which, in turn, is more susceptible to infection, forming a vicious circle and aggravating the disease.

### Antibiotic Therapies in Patients With AD and Their Models

The main effects of antibiotic therapies are eliminating and avoiding bacterial colonization in human body *via* their directly killing bacteria or indirectly changing the composition of gut microbiota that have been evidenced to associate with AD pathogenesis (Angelucci et al., 2019). Therapies with antibiotics could alter behavior and brain chemistry in both humans and animals (Fröhlich et al., 2016; Neuman et al., 2018). Antibiotic therapy lowered obviously amounts of microglia and astrocyte accumulation around Aβ plaques in the hippocampus, and reduced insoluble Aβ plaques in aged APPSWE/PS1DeltaE9 transgenic mice of the AD model, indicating that antibiotic therapy was effective in AD mice (Minter et al., 2017). However, the therapy has not yet widely investigated in patients with AD, because it is unknown whether there are safe antibiotics available (Panza et al., 2019). Since the patients with AD infected with *H. pylori*, receiving antibiotic treatment, displayed neuropsychiatric symptoms, such as anxiety, psychosis, and delirium in two studies (Loeb et al., 2004; Molloy et al., 2013), these neuropsychiatric symptoms did not appear in the general subjects (Neufeld et al., 2017). Similarly, the harmful effects of antibiotics on APP/PS1 transgenic mice manifested as enhanced neuroinflammation and pro-inflammatory cytokine levels and deteriorated disease (Minter et al., 2016). Ampicillin increased rat serum corticosterone related to memory dysfunctions and a lowered brain-derived neurotrophic factor (BDNF) in the hippocampus, the features of AD pathology, and deteriorated the anxiety-like behavior and impaired spatial memory in rats (Fröhlich et al., 2016). The clinical and experimental studies highlight that the results with antibiotic treatment in AD are controversial. Also, there were some adverse consequences after antibiotics treatments. It is currently difficult to determine the effect of antibiotics on AD. Therefore, the future studies on therapeutic trials with antibiotics in AD are required.

### Interferon and Other Antivirus Therapies in AD and Their Animal Models

Activated IFN type I, a viral infection inhibitor, and stimulated gene (ISG)-expressing microglia were found in Aβ plaques in an age-dependent manner in AD mice. Injection of recombinant IFN-β into mouse brain caused complement C3-dependent synapse loss *in vivo*. In turn, a blockade of the IFN receptor lightened microgliosis and synapse loss in AD mice. ISG-expressing microglia were also detected in postmortem brains of patients with AD, indicating that the IFN pathway was markedly upregulated in AD and close related to disease severity and complement activation. Therefore, IFN critically promotes neuropathogenic processes (Roy et al., 2020). Valacyclovir, as an antivirus drug, has been conducted for the clinical trial in 130 patients with mild AD (65 patients in valacyclovir and in placebo groups, respectively) in a randomized, double-blind, 78-week phase II to decide valacyclovir to apply in treatment for AD *via* its anti-HSV effect (Devanand et al., 2020). There is no doubt when completion of the trial and the effect of valacyclovir on AD might be clear. The anti-infection therapies in AD animal models and patients are summarized in Table 1.

**TABLE 1:** Anti-infection therapies in AD animal models and patients.

Pre-clinical and clinical studies	Anti-infection therapies targeting pathogens	Therapeutic results	References
APPSWE/PS1ΔE9 mice	Akkermansia; Lachnospiraceae	Shifting gut microbial composition and diversity Brain Aβ deposition↓ Brain soluble Aβ↑ Blood: CCL11, CXCL16, LIX, TIMP-1 and NFαR1↑	Minter et al., 2016
APPSWE/PS1ΔE9 mice	Lachnospiraceae and S24-7	Brain Aβ deposition↓ Microglia and astrocyte accumulation around amyloid plaques↓ Blood: Foxp3+ T-regulatory cells ↑	Minter et al., 2017
APP/PS1 mice	Eubacteria, Roseburia, Clostridium	Spatial memory↑ Hippocampus Aβ plaques↓ The numbers of microglia in hippocampus ↓ OOG1 in the hippocampus↓	Abraham et al., 2019
Wistar rats intrahippocampal injection of Aβ1–42	Coliform, bifidobacterial, lactobacilli	Learning and memory abilities↑ MDA in the hippocampus ↓ SOD in the hippocampus ↓	Athari Nik Azm et al., 2018
AD patients	*Helicobacter pylori*	Cognitive and functional status parameters ↑	Kountouras et al., 2009
AD patients	*Chlamydia pneumoniae*	Cognition ↑	Loeb et al., 2004
AD patients	*Faecalibacterium prausnitzii*	Fecal zonulin concentrations ↓ *Faecalibacterium prausnitzii* ↑ Serum kynurenine concentrations ↑	Leblhuber et al., 2018
AD patients	Herpes simplex virus	Cognitive function ↑ Amyloid and tau accumulations in the brain ↓	Devanand et al., 2020
AD patients	*Lactobacillus acidophilus*, *Lactobacillus casei*, *Bifidobacterium bifidum*, *Lactobacillus fermentum*	MMSE score↑ Hs-CRP, HOMA-IR, HOMA-B, and MDA in blood↓ QUICKI↑	Akbari et al., 2016

*OOG1, 8-oxoguanine DNA glycosylase-1; MDA, Malondialdehyde; SOD, Superoxide dismutase; MMSE, Mini-mental state examination; Hs-CRP, High sensitivity C-reactive protein; HOMA-IR, Homeostatic model of assessment for insulin resistance; QUICKI, Quantitative insulin sensitivity check index.*

Up to now, no study has confirmed clearly that anti-microbe treatment in AD is beneficial and effective, which is needed to evidence in the future studies.

## Conclusion

Infections mainly caused by *P. gingivalis*, *H. pylori*, HIV, and HSV-1, and chronic inflammation caused by infections as the most likely etiologies contribute to the pathogenesis of AD. It is linked to rising pro-inflammatory and lower anti-inflammatory response, and to promote Aβ plaques deposits and hyperphosphorylated tau protein formation in brain of AD, which damages brain health and causes the pathological changes in AD. Infection may be a key pathogenic factor in development and progression of AD, which has been evidenced through the experimental and preclinical studies currently. However, the results obtained are controversial, and it is still difficult to determine their causal relationship between infection and AD so far. By listing a large number of results and in-depth discussions in the present review, we should pay attention to the pathogenic role of infection in AD; albeit infection may not necessarily cause AD directly, it is a trigger or promoter in development of AD definitely. The further study and randomized controlled trials in humans are essential to define the impact of infection on AD. Discovery of novel anti-infectious compounds and determining the time window application of antibacterial or antiviral intervention are more hopeful for the future clinical treatment strategy of AD.

## Author Contributions

MS and CL prepared the manuscript. XT and FC provided views and revised the manuscript. JZ designed the framework of the manuscript and prepared and finalized the manuscript. All authors agreed to approved the final manuscript.

## Conflict of Interest

The authors declare that the research was conducted in the absence of any commercial or financial relationships that could be construed as a potential conflict of interest.

## Abbreviations

AD: Alzheimer’s disease
A β: amyloid β
*P. gingivalis*: *Porphyromonas gingivalis*
*H. pylori*: *Helicobacter pylori*
*C. albicans*: *Candida albicans*
*C. neoformans*: *Cryptococcus neoformans*
HIV: human immunodeficiency virus
HSV-1: herpes simplex virus type 1
HHV-6: human herpes virus-6
EBV: Epstein–Barr virus
COVID-19: coronavirus 2019
CNS: central nervous system
IL: interleukin
TNF- α: tumor necrosis factor-alpha
NO: nitric oxide
Th: T helper
CSF: cerebrospinal fluid
APOE: apolipoprotein
KO: knockout
Cat: cathepsin
NF- κ B: nuclear factor kappa-B
WT: wild type
VaD: vascular dementia
PD: Parkinson’s disease
MMSE: mini-mental state examination
TTV: Torque teno virus
AIDS: acquired immune deficiency syndrome
EVs: extracellular vesicles
BBB: blood-brain barrier
NFT: neurofibrillary tangles
Tat: transcription
HAND: HIV-associated neurocognitive disorder
MCI: mild cognitive impairment
ER: endoplasmic reticulum
UPR: unfolded protein response
RNA-seq: RNA sequencing
CVD: cardiovascular disease
PrPSc: prion protein scrapie
PrP^C^: cellular prion protein
BDNF: brain-derived neurotrophic factor
IFN: type I interferon
ISG: interferon-stimulated gene.
